# Clinical features and risk factors for ICU admission in COVID-19 patients with cardiovascular diseases

**DOI:** 10.14336/AD.2020.0622

**Published:** 2020-07-23

**Authors:** Feng He, Yibo Quan, Ming Lei, Riguang Liu, Shuguang Qin, Jun Zeng, Ziwen Zhao, Na Yu, Liuping Yang, Jie Cao

**Affiliations:** ^1^Guangzhou First People's Hospital, The Second Affiliated Hospital of South China University of Technology, Guangzhou, China.; ^2^Guangzhou Eighth People's Hospital, Guangzhou Medical University, Guangzhou, China.

**Keywords:** COVID-19, cardiovascular disease, clinical features, risk factors, intensive care unit

## Abstract

Previous studies on coronavirus disease 2019 (COVID-19) have focused on the general population. However, cardiovascular disease (CVD) is a common comorbidity that has rarely been investigated in detail. This study aims to describe clinical characteristics and determine risk factors for intensive care unit (ICU) admission of COVID-19 patients with CVD. In this retrospective cohort study, we included 288 adult patients with COVID-19 in Guangzhou Eighth People's Hospital from January 15, 2020 to March 10, 2020. Demographic characteristics, laboratory results, radiographic findings, complications, and treatments were recorded and compared between CVD and non-CVD groups. A binary logistic regression model was used to identify risk factors associated with ICU admission for infected patients with underlying CVD. COVID-19 patients in the CVD group were older and had higher levels of troponin I (TnI), C-reactive protein (CRP), and creatinine. They were also more prone to develop into severe or critically severe cases, receive ICU admission, and require respiratory support treatment. Multivariate regression analysis showed that the following were risk factors for ICU admission in COVID-19 patients with CVD: each 1-year increase in age (odds ratio (OR), 1.08; 95% confidence interval (CI), 1.02-1.17; p = 0.018); respiratory rate over 24 times per min (OR, 25.52; 95% CI, 5.48-118.87; p < 0.0001); CRP higher than 10 mg/L (OR, 8.12; 95% CI, 1.63-40.49; p = 0.011); and TnI higher than 0.03 μg/L (OR, 9.14; 95% CI, 2.66-31.43; p < 0.0001). Older age, CRP greater than 10 mg/L, TnI higher than 0.03 μg/L, and respiratory rate over 24 times per minute were associated with increasing odds of ICU admission in COVID-19 patients with CVD. Investigating and monitoring these factors could assist in the risk stratification of COVID-19 patients with CVD at an early stage.

The coronavirus disease 2019 (COVID-19) has been identified as an ongoing pandemic in the globe and is caused by the Severe Acute Respiratory Syndrome Coronavirus-2 (SARS-CoV-2) [1]. Previous studies have mainly focused on the general population. However, as a common comorbidity, cardiovascular disease (CVD) has been rarely investigated in detail.

Recent data have shown a significant percentage of CVD among COVID-19 patients, which raises many questions about the higher susceptibility of patients with these comorbidities to the SARS-CoV-2 infection, as well as the role of CVD in the progression and prognosis of COVID-19 patients [2]. Increasing numbers of confirmed cases and mortality rates of COVID-19 are associated with underlying CVD [3-5]. Data from the Chinese Center for Disease Control and Prevention demonstrated that the overall case-fatality rate (CFR) of COVID-19 was 2.3%, whereas CFR was elevated in preexisting chronic conditions, especially reaching 10.5% in patients with underlying CVD [6]. Additionally, respiratory syndrome was the main clinical manifestation in COVID-19 patients, but 10-17% of hospitalized patients suffered from cardiac injury [4, 5, 7]. However, it is currently unclear whether COVID-19 patients who have underlying CVD and develop cardiac injuries during hospitalization have worse in-hospital outcomes. Therefore, the present study aimed to describe clinical characteristics and identify risk factors for the intensive care unit (ICU) admission of COVID-19 patients with CVD.

## MATERIALS AND METHODS

In this retrospective cohort study, 288 hospitalized patients with laboratory-confirmed COVID-19 were enrolled in Guangzhou Eighth People's Hospital (Guangzhou, China) from January 15, 2020 to March 10, 2020. This included 85 (29.51%) patients with preexisting CVD. A confirmed case was defined as having a positive result via real-time reverse transcription polymerase chain reaction assay or high-throughput sequencing of nasopharyngeal swab specimens. The patients were diagnosed and classified as four clinical types (i.e., mild, common, severe, and critically severe), according to the Chinese Clinical Guidance for COVID-19 Pneumonia Diagnosis and Treatment (7th edition) (http://kjfy.meetingchina.org/msite/news/show/cn/3337.html). CVD was defined as the clinical diagnosis of coronary heart disease, cerebrovascular disease, peripheral arterial disease, rheumatic or congenital heart diseases, or venous thromboembolism [8]. Informed consent was obtained from all patients enrolled.

Information extracted from clinical electronic records included demographic data, comorbidities, symptoms and signs, laboratory findings, complications, and chest computed tomography (CT) scans. To analyze risk factors associated with the ICU admission of 85 COVID-19 patients with CVD, univariable and multivariable logistic regression analyses were performed. The multivariable logistic regression model was constructed using only variables with a significant *p*-value (<0.05) in univariate logistic regression analysis. A *p-*value less than 0.05 was considered statistically significant. The SPSS (Statistical Package for the Social Sciences) 25.0 software was used for all analyses.

## RESULTS

### Baseline characteristics

From January 15, 2020 to March 10, 2020, 288 adult patients with COVID-19 were hospitalized in Guangzhou Eighth People's Hospital, and 85 (29.5%) of these patients also had CVD. The median age of all patients was 48.5 years (interquartile range, 34.3-62.0), and 45.5% (131/288) were male (Table 1). Fever (n = 201, 69.8%) and cough (n = 163, 56.6%) were the most common symptoms at the onset of illness for all patients, followed by sore throat (n = 67, 23.3%) and fatigue (n = 43, 14.9%) (Table 1). Hypertension (n = 84; 29.2%) and diabetes (n = 24, 8.3%) were common comorbidities for all patients. Compared to COVID-19 patients without CVD, infected patients with CVD were older and more likely to have hypertension and diabetes, suffer respiratory rates exceeding 24 times per min, and require respiratory support treatment (Table 1).

**Table 1:** Clinical features of COVID-19 patients with CVD.

Characteristic	Total (n=288)	non-CVD (n=203)	CVD (n=85)	*p* value
**Demographics**				
Age, median (IOR), years	48.5 (34.3, 62.0)	43.0 (32.0, 57.0)	62.0 (53.3, 67.0)	<0.0001
Male, n (%)	131 (45.5)	85 (41.9)	118 (58.1)	0.057
Comorbidities				
Hypertension	84 (29.2)	1 (0.5)	83 (97.6)	<0.0001
Diabetes	24 (8.3)	8 (3.9)	16 (18.8)	<0.0001
Chronic kidney disease	8 (2.8)	3 (1.5)	5 (5.9)	0.038
COPD	5 (1.7)	1 (0.5)	4 (4.8)	0.012
Cerebrovascular disease	8 (2.8)	0 (0.0)	8 (9.4)	<0.0001
Chronic liver disease	10 (3.5)	6 (3.0)	4 (4.7)	0.459
Malignancy	6 (2.1)	4 (2.0)	2 (2.4)	0.836
**Signs and symptoms**				
Fever	201 (69.8)	146 (71.9)	55 (64.7)	0.224
Chill	55 (19.1)	44 (21.7)	11 (12.9)	0.085
Cough	163 (56.6)	110 (54.2)	53 (62.4)	0.202
Rhinorrhea	18 (6.3)	11 (5.4)	7 (8.2)	0.368
Sore throat	67 (23.3)	44 (21.7)	23 (27.1)	0.324
Fatigue	43 (14.9)	34 (16.7)	9 (10.6)	0.181
Myalgia or arthralgia	35 (12.2)	25 (12.3)	10 (11.8)	0.896
Headache	26 (9.0)	21 (10.3)	5 (5.9)	0.228
Nausea	28 (9.7)	22 (10.8)	6 (7.1)	0.324
Diarrhea	11 (3.8)	10 (4.9)	1 (1.2)	0.13
Respiratory rate ≥24 breaths per min	19 (6.6)	8 (3.9)	11 (12.9)	0.005
Oxygen support				
None	88 (30.6)	65 (32.0)	23 (27.1)	0.405
Normal-flux	184 (63.9)	131 (64.5)	53 (62.4)	0.725
High-fux	16 (5.6)	7 (3.4)	9 (10.6)	0.016
Non-invasive mechanical ventilation	32 (11.1)	15 (7.4)	17 (20)	0.002
Unilateral pneumonia	241 (83.7)	165 (81.3)	76 (89.4)	0.089
Bilateral pneumonia	31 (10.8)	25 (12.4)	6 (7.1)	0.185
**Laboratory variables**				
White blood cell, × 10^9^/L	5.20 (4.14, 6.44)	5.01(4.03, 6.22)	5.64 (4.42, 7.18)	0.271
White blood cell count, × 10^9^/L (No. (%))				0.109
≤4	62 (21.5)	49 (24.1)	13 (15.3)	
4-10	216 (75.0)	149 (73.4)	67 (78.8)	
>10	10 (3.5)	5 (2.5)	5 (5.9)	
Lymphocyte count, × 10^9^/L	1.42 (1.04, 1.96)	1.45 (1.06, 2)	1.31 (0.9, 1.84)	0.373
Lymphocyte count, × 10^9^/L (No. (%))				0.751
<1.1	91 (31.6)	63 (31.0)	28 (32.9)	
Platelet count, × 10^9^/L	194.5 (158, 247)	196 (160, 248)	190 (153, 240.5)	0.762
Hemoglobin, g/L	135.5 (123, 147)	135 (123, 146)	136 (121.5, 147.5)	0.782
Procalcitonin, ng/mL	0.13 (0.04, 32.6)	0.09 (0.03, 32.6)	0.30 (0.05, 34.9)	0.314
C-reactive protein, mg/L	9 (8, 22.7)	9 (8, 18.04)	13.18 (8, 37.5)	0.004
C-reactive protein, mg/L (No. (%))				0.001
≤10	175 (60.8)	136 (67)	39 (45.9)	
>10	113 (39.2)	67 (33)	46 (54.1)	
Troponin I, μg/L (No. (%))				0.002
≤0.03	168 (88.4)	116 (93.5)	52 (78.8)	
>0.03	22 (11.6)	8 (6.5)	14 (21.2)	
BNP, μg/L	35 (13, 117.5)	18.5 (9, 74.5)	45 (22, 179)	0.177
Creatine kinase, U/L	52 (36, 80)	54 (38, 80)	48.5 (28.75, 82.5)	0.385
Creatinine, μmol/L	61.8 (50.2, 76.58)	58.8 (48.1, 74.9)	66.9 (52.1, 78)	0.001
ATL, U/L	22.45 (14.3, 34.5)	22.4 (14, 34.4)	22.5 (15.9, 34.9)	0.317
AST, U/L	18.35 (14.9, 25.63)	17.7 (13.7, 24.5)	19.4 (16.4, 27.4)	0.105
**Complications**				
Acute cardiac injury	22 (11.6)	8 (6.5)	14 (21.2)	0.020
ARDS	3 (1.0)	1 (0.5)	2 (2.4)	0.156
Acute kidney injury	5 (1.7)	2 (1.0)	3 (3.5)	0.132
Clinical Types ^a^ (Severe/Critically severe)	30 (10.4)	15 (7.4)	15 (17.6)	0.009
ICU Admission	27 (9.4)	12 (5.9)	15 (17.6)	0.002

Data are presented as median [interquartile range] or number (percent).

Abbreviations: CVD, cardiovascular disease; COPD, chronic obstructive pulmonary disease; BNP, brain natriuretic peptide; ALT, alanine transaminase; AST, aspartate transaminase; ARDS, acute respiratory distress syndrome; ICU, intensive care unit; OR, odds ratio; CI, confidence interval.

a Clinical Types (Severe/Critically severe) was based on the Chinese Clinical Guidance for COVID-19 Pneumonia Diagnosis and Treatment (7th edition) published by National Health Commission of China.

### Laboratory and radiological findings

**Figure 1.**
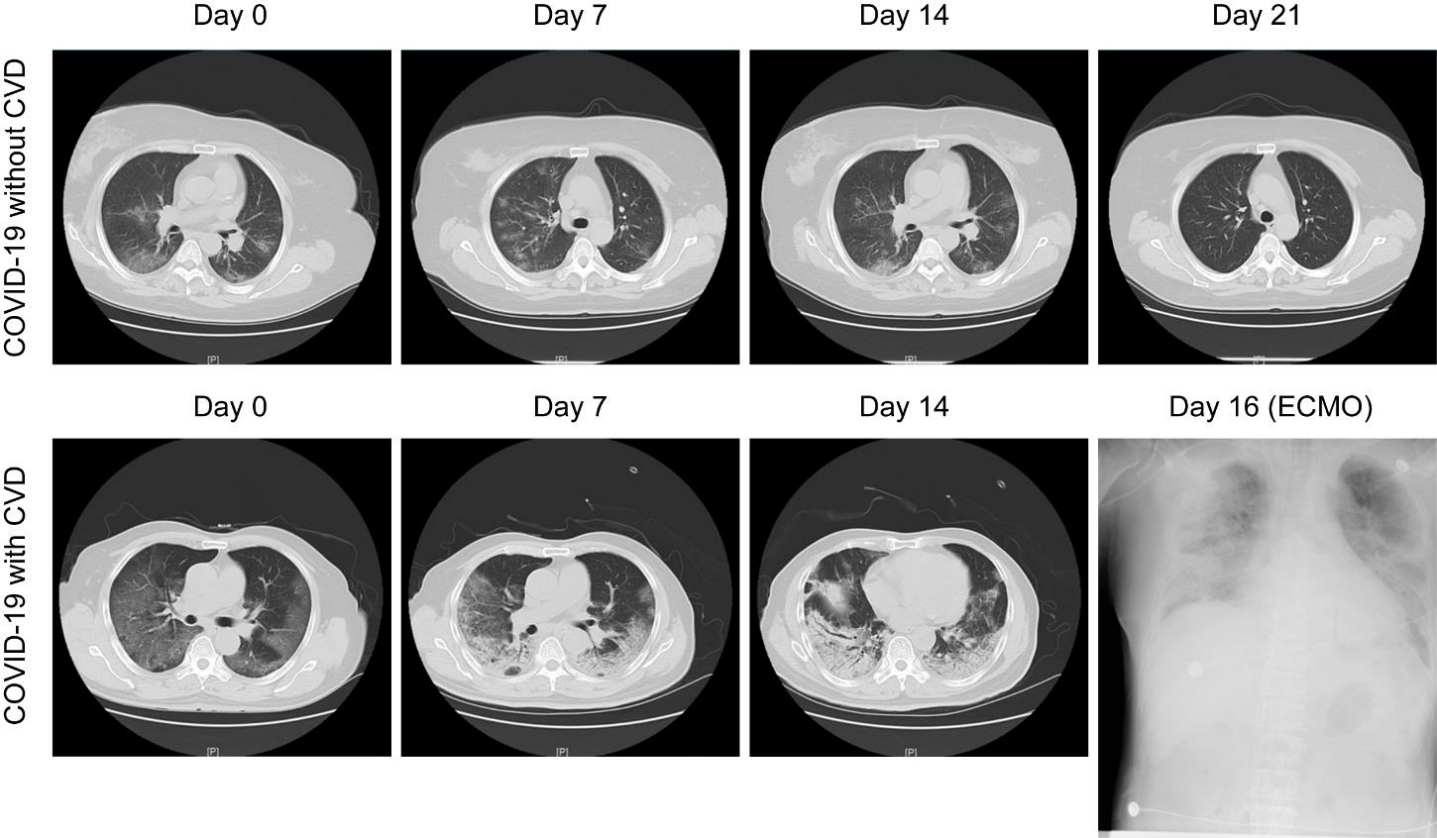
**Representative chest computed tomography (CT) images of COVID-19 pneumonia in a non-cardiovascular disease (CVD) case and a CVD case**. Top panel: A 60-year-old man with COVID-19, but not CVD: chest CT images showed ground-glass opacity (GGO) and patchy consolidation with peripheral and subpleural distribution, which had been absorbed at 21 days after hospitalization with treatment. Bottom panel: A 65-year-old man with both COVID19 and CVD: chest CT images showed diffusely subpleural consolidation with a crazy-paving pattern. Diffuse shadowing and consolidation were seen on chest radiography after intensive care unit (ICU) admission with extracorporeal membrane oxygenation (ECMO) support at day 16.

Sixty-two (21.5%) patients had low white blood cell counts, and lymphopenia (lymphocyte count < 1.1 × 10^9^/L) occurred in 91 (31.6%) patients (Table 1). Compared to COVID-19 patients without CVD, C-reactive protein (CRP), troponin I (TnI), and serum creatinine were significantly increased in COVID-19 patients with CVD (Table 1). Thirty-one (10.8%) patients with bilateral pneumonia were identified by chest radiological examination, and 89.4% of them had CVD (Table 1). Notably, COVID-19 patients without CVD had relatively favorable manifestations of chest CT images, including bilateral multifocal ground-glass opacities and patchy consolidations (Fig. 1, top panel). In contrast, COVID-19 patients with CVD showed a rapid and worsening radiographic progression, evidenced by diffusely subpleural consolidation with crazy-paving patterns on chest CT images (Fig. 1, bottom panel). Moreover, COVID-19 patients with CVD were more likely to have acute cardiac injury, develop into severe or critically severe cases, and receive ICU admission (Table 1).

**Table 2:** Risk factors of ICU admission for COVID-19 patients with CVD.

		Univariable OR (95% CI)	*p* value	Multivariable OR (95% CI)	*p* value
Age, years		1.09 (1.02-1.15)	0.010	1.08 (1.02-1.17)	0.018
Female sex (vs. male)		0.24 (0.06-0.91)	0.036	0.18 (0.29-1.06)	0.058
Diabetes		1.76 (0.48-6.46)	0.396	..	..
Chronic kidney disease		3.44 (0.52-22.64)	0.199	..	..
COPD		1.72 (0.17-17.83)	0.650	..	..
Cerebrovascular disease		1.64 (0.30-9.05)	0.57	..	..
Fever		1.63 (0.47-5.63)	0.444	..	..
Cough		2.83 (0.73-10.93)	0.132	..	..
Respiratory rate ≥24 breaths per min		37.77 (5.52-258.28)	<0.0001	25.52 (5.48-118.87)	<0.0001
White blood cell count, ×10^9^/L					
	≤4	0	0.999	..	..
	4-10	1(ref)	..	..	..
	>10	2.77 (0.42-18.31)	0.291	..	..
Lymphocyte count, ×10^9^/L					
	<1.1	5.78 (1.74-19.18)	0.004	0.47 (0.15-1.52)	0.210
	≥1.1	1(ref)	..	1(ref)	..
Procalcitonin, ng/mL					
	<0.05	1(ref)	..	1(ref)	..
	0.05-10	4.07 (0.78-21.27)	0.096	4.13 (0.42-40.77)	0.225
	>10	1.31 (0.22-7.87)	0.768	0.48 (0.03-7.44)	0.602
C-reactive protein, mg/L					
	<10	1(ref)	..	1(ref)	..
	≥10	24.57 (5.69-106.13)	<0.0001	8.12 (1.63-40.49)	0.011
Troponin I, ug/L					
	≤0.03	1(ref)	..	1(ref)	..
	>0.03	14.72 (5.03-43.13)	<0.0001	9.14 (2.66-31.43)	<0.0001
BNP, 100 μg/L					
	≤100	1(ref)	..	..	..
	>100	60 (5.47-658.76)	0.001	..	..
Creatinine, μmol/L					
	≤106	1(ref)	..	1(ref)	..
	>106	5.31 (1.25-22.60)	0.024	2.00 (0.18-22.08)	0.572

OR, odds ratio; CI, confidence interval; COPD, chronic obstructive pulmonary disease; BNP, brain natriuretic peptide.

### Risk factors for ICU admission of COVID-19 patients with CVD

In the univariate logistic regression analysis, we found that higher odds of ICU admission were related to older age, respiratory rate over 24 breaths per min, lymphopenia, and increased levels of CRP, TnI, brain natriuretic peptide, and creatinine (Table 2). In the multivariable logistic regression analysis, we revealed that each 1-year increase in age (odds ratio (OR), 1.08; 95% confidence interval (CI), 1.02-1.17; *p* = 0.018), a respiratory rate over 24 breaths per min (OR, 25.52; 95% CI, 5.48-118.87; *p* < 0.0001), CRP higher than 10 mg/L (OR, 8.12; 95% CI, 1.63-40.49; *p* = 0.011), and TnI higher than 0.03 μg/L (OR, 9.14; 95% CI, 2.66-31.43; *p* < 0.0001) were risk factors for the ICU admission of COVID-19 patients with CVD (Table 2).

## DISCUSSION

We reported a retrospective cohort study of 288 adult hospitalized patients with COVID-19, including 85 patients with underlying CVD. Compared with the non-CVD group, COVID-19 patients in the CVD group were older and had higher levels of TnI, CRP, and creatinine. These patients were also more likely to develop into severe or critically severe cases, receive ICU admission, and require respiratory support treatment. In particular, multivariable regression revealed that older age, TnI levels greater than 0.03 μg/L, CRP levels greater than 10 mg/L, and a respiratory rate over 24 times per minute were associated with increasing odds of ICU admission in COVID-19 patients with underlying CVD.

A recent meta-analysis assessed the prevalence of comorbidities of COVID-19 patients and suggested that CVD may be a risk factor for developing severe cases [9]. Consistently, our cohort study provided reliable evidence that COVID-19 patients with underlying CVD were more likely to develop into severe or critically severe cases, require respiratory support treatment, and eventually receive ICU admission. Early reports have suggested that older adults have elevated rates of COVID-19-associated hospitalization and increased mortality rates of hospitalized patients with COVID-19 [5]. In our study, COVID-19 patients with CVD were older and more prone to be transferred into the ICU. The mechanism of this association is still unclear, but is increasingly being recognized with dysfunctional immune systems [10]. A previous study reported that aging patients experience a marked decline of cell-mediated and humoral immune function, which could bring an impaired antiviral response of the host and result in an uncontrolled viral replication process [11]. Moreover, a retrospective analysis demonstrated that a large area of lung injury (≥50%) in severe cases is closely correlated with increased levels of inflammatory cytokines such as interleukin-6, which promotes inflammation and is gradually upregulated with increased age [11, 12]. Therefore, age-dependent immune defects and dysregulated pro-inflammatory response could be characteristics of aging patients, thereby potentially leading to more severe outcomes, such as ICU admission. Furthermore, our study expands the observations showing that COVID-19 patients with CVD had higher levels of CRP. Particularly, a CRP level greater than 10 mg/L suggested a higher risk for ICU admission in COVID-19 patients with CVD. Given that CRP has capabilities of complement activation and phagocytosis enhancement, the plausible mechanism is related to a severe inflammatory cascade, which could induce the cytokine storm and lead to multiple organ dysfunction [3, 7].

Our study found an association of TnI with adverse outcomes of ICU admission in COVID-19 patients with underlying CVD. A recent study of 187 cases also suggested that COVID-19 patients with CVD are more likely to experience acute cardiac injury and be associated with fatal outcomes [13]. Moreover, patients with CVD and normal TnI levels had a relatively favorable prognosis [13]. Thus, it is necessary to evaluate TnI levels in COVID-19 patients with CVD for preventing cardiac complications and providing early interventions. The mechanism of this cardiac involvement remains under investigation. Given that angiotensin-converting enzyme 2 (ACE2) is a functional receptor of SARS-CoV-2 entry into the host cell and is widely expressed not only in the lungs but in the heart, there could be a potential possibility of direct damage to myocytes, which causes an elevated TnI level [5, 14].

Drugs for CVD treatment may also affect the outcome of COVID-19. For instance, ACE inhibitors (ACEI) and angiotensin-receptor blockers (ARB) are common drugs for cardiovascular disorders, such as hypertension. Some data suggest that the use of ACEI/ARB could upregulate ACE2 expression, thereby increasing susceptibility to the virus and also inducing acute cardiac injury in those with underlying CVD [10, 14, 15]. In addition, ACE2 has another function of protecting the lungs by downregulating the level of angiotensin II, which is a potent pro-inflammatory agent in lung injury, and ACEI/ARB treatment may enhance this protection by increasing the secretion of ACE2 [10]. Thus, the use of ACEI/ARB shows a controversial role in promoting viral infection and cardiac complication while suppressing pathogenic inflammation, especially in COVID-19 patients with underlying CVD. Of note, a recent retrospective study based on 1128 hospitalized COVID-19 patients with hypertension suggested a lower risk of all-cause mortality among those receiving ACEI/ARB treatment when compared with ACEI/ARB non-users [16], which provided evidence to support the continued use of ACEI/ARB in COVID-19 patients.

The SARS-Cov2 invasion of lungs through ACE2 results in severe respiratory dysfunction and hypoxemia, which are the main causes of mortality of COVID-19 [3]. In the present study, a respiratory rate over 24 times per minute was associated with higher odds of ICU admission. We proposed a likely explanation that ACE2 not only directly damages myocytes, but also induces hypoxemia-related myocardial injury, thus increasing risk in COVID-19 patients, especially those with underlying CVD [13, 14]. Therefore, the assessment of respiratory status is also extremely important in screening high-risk patients.

In conclusion, we determined that older age, a respiratory rate over 24 breaths per minute, and elevated TnI and CRP levels are risk factors for ICU admission in COVID-19 patients with CVD. Investigating and monitoring these factors could assist in the risk stratification of COVID-19 patients with CVD, so that timely and aggressive interventions can be implemented at an early stage. It would also provide significant experience and references for global anti-epidemic work in patients with CVD.
